# The therapy of insulin resistance in other diseases besides type 2 diabetes

**DOI:** 10.1007/s40519-014-0139-y

**Published:** 2014-07-29

**Authors:** Laura Pala, Valeria Barbaro, Ilaria Dicembrini, Carlo Maria Rotella

**Affiliations:** 1 Endocrine Unit, Department of Biomedical Experimental and Clinical Sciences, University of Florence, Careggi University Hospital, Florence, Italy; 2 Department of Biomedical Experimental and Clinical Sciences, Obesity Agency, University of Florence, Careggi University Hospital, Viale Pieraccini 6, 50134 Florence, Italy; 3 Department of Biomedical Experimental and Clinical Sciences, Diabetes Agency, University of Florence, Careggi University Hospital, Florence, Italy

**Keywords:** Metformin, Insulin resistance, PCOS, NAFLD, Obesity

## Abstract

Insulin resistance is a clinical condition shared by many diseases besides type 2 diabetes (T2DM) such as obesity, polycystic ovary syndrome (PCOS) and non-alcoholic fatty liver disease (NAFLD). Experimental evidence, produced over the years, suggests that metformin has many benefits in the treatment of these diseases. Metformin is a first-line drug in the treatment of overweight and obese type 2 diabetic patients, offering a selective pathophysiological approach by its effect on insulin resistance. Moreover, a number of studies have established the favorable effect of metformin on body weight, not only when evaluating BMI, but also if body mass composition is considered, through the reduction of fat mass. In addition, it reduces insulin resistance, hyperinsulinemia, lipid parameters, arterial hypertension and endothelial dysfunction. In particular, a new formulation of metformin extended-release (ER) is now available with different formulation in different countries. Metformin ER delivers the active drug through hydrated polymers which expand safe uptake of fluid, prolonging gastric transit and delaying drug absorption in the upper gastrointestinal tract. In addition, Metformin ER causes a small, but statistically significant decrease in BMI, when added to a lifestyle intervention program in obese adolescents. Because of the suggested benefits for the treatment of insulin resistance in many clinical conditions, besides type 2 diabetes, the prospective exists that more indications for metformin treatment are becoming a reality.

## Introduction

### Insulin resistance (IR) and metformin

During the postprandial state, insulin secretion by the pancreatic β cells controls nutrient homeostasis by promoting anabolic processes in many tissues. During the fasting state, insulin secretion decreases and tissues are acted upon by counter-regulatory hormones, in favor of using fatty acids largely derived from adipocyte lipolysis for the generation of ATP and maintenance of glucose homeostasis. The substrate preferences for metabolic adaptation, during the transit from the fasting to the postprandial state, are tightly controlled by insulin under physiological conditions [1]. This adaptive transition reflects the action of insulin in insulin-responsive organs, while it is largely blunted in organs with IR preceding the development of type 2 diabetes (T2DM) [2]. IR produces different effects in different organs and tissues. In central nervous system (CNS), IR causes obesity, since in humans, appetite is increased by the action of insulin in the CNS and the current data indicate that neuronal insulin signaling is required for both body weight control and glucose homeostasis [3]. IR in adipose tissue is responsible for hyperlipidemia, in fact, in normal condition, insulin promotes fat cell differentiation, enhances adipocyte glucose uptake and inhibits adipocyte lipolysis; when insulin action is deficient in adipose tissue, adipocyte development is tampered and lipids are not generated from carbohydrates for storage. Adipose tissue is also an endocrine organ, secreting cytokines and hormones, and fat expansion disrupts a proper balance of cytokine and hormone generation, promoting insulin resistance [4]. Insulin stimulates the synthesis of glycogen, lipids and protein in the liver and suppresses hepatic glucose production by inhibiting gluconeogenesis, thus controlling blood glucose and lipid homeostasis, therefore hepatic insulin resistance generates hyperglycemia [5]. IR in pancreas impairs β cell regeneration: recent studies have shown that insulin enhances glucose-stimulated insulin secretion in healthy humans [6], however, whether insulin may have a direct autocrine action on β cells in promoting insulin secretion is unclear [7]. Skeletal muscle is an important fuel storage tissue for glucose uptake, converting it to glycogen and triglycerides; this process, stimulated by insulin, is impaired in the IR condition [8]. Cardiac insulin resistance promotes heart failure: the heart is an insulin-responsive and energy-consuming organ that requires a constant fuel supply to maintain intracellular ATP levels for myocardial contraction [9]. IR in vascular endothelium promotes hypertension and disrupts glucose homeostasis [10]. IR in bone impairs glucose homeostasis acting on the synthesis of osteocalcin [11].

Metformin because of its efficacy, security profile, and benefic cardiovascular and metabolic effects is the first glucose-lowering agent of choice in the treatment of T2DM together with lifestyle modifications [12]. Metformin decreases hepatic glucose output lowering fasting glycaemia and increases glucose uptake in peripheral tissues. Interest in the therapeutic use of metformin has been sparked by the recognition of its pleiotropic actions on several tissues, which are affected by IR and hyperinsulinemia. Although the liver is the primary target organ, metformin acts on a variety of tissues, namely skeletal muscles, adipose tissue, endothelium and the ovary [13].

## Metformin and other drugs for the IR therapy in other diseases besides type 2 diabetes

Experimental evidence suggests that metformin may be useful in some clinical conditions different from T2DM [14]. Metformin offers a selective pathophysiological approach by its effect on IR. It has been shown in a number of studies that it improves clinical outcomes in type 2 diabetic patients via multiple biological effects: it has been shown, also, to retain platelet antiaggregating effects, to reduce the rate of formation of advanced glycation end products (AGEs) and to decrease the cellular oxidative reactions, thus demonstrating its vascular protective effect. A number of studies have established the favorable effect of metformin on body weight, IR, hyperinsulinemia, lipid parameters, arterial hypertension, fibrinolysis, and endothelial dysfunction. On this basis, metformin appears to have a broad set of pharmacological properties, making the drug potentially applicable even in non-diabetic situations such as obesity, extreme insulin resistance with acanthosis nigricans, polycystic ovary syndrome (PCOS) and the non-alcoholic fat liver disease (NAFLD). Metformin has been demonstrated in the Diabetes Prevention Program to have a role in preventing the conversion of IGT to T2DM and to be a drug with multiple therapeutic effects far beyond its effect on lowering blood glucose in T2DM [15].

### Obesity

After binding to its receptor and activating the β-subunit, insulin is faced with two divergent pathways: one is phosphatidylinositol 3-kinase (PI 3-K) dependent, while the other is dependent upon activation of mitogen-activated protein kinase (MAP-K). The former mediates most gluco-metabolic and anti-apoptotic effects; the latter is linked to liposynthetic, proliferative and mitogenic effects. In obese patients, especially with T2DM, only the PI 3-K, but not the MAP-K, is resistant to insulin stimulation: hence IR is better defined as the gluco-metabolic insulin resistance. The resulting “compensatory hyperinsulinemia” is an unsuccessful attempt to overcome the inhibition of the gluco-metabolic pathway at the price of unopposed stimulation of the MAP-K pathway and the administration of exogenous insulin might worsen the metabolic dysfunction. As the preferential activation of the MAP-K pathway in insulin-resistant patients has atherogenic and mitogenic properties, this may lead to atherosclerosis and cancer. Metformin, in addition, may carry out direct protective action on human β cells, inasmuch as it improves both primary and secondary endpoints through selective inhibition of fatty acyl oxidation [16]. Many studies have demonstrated that metformin is associated with weight reduction in adults and the prevention or delay of T2DM onset, in those individuals who are at increased risk. However, consensus is lacking on intervention strategies aimed at reducing this risk, as reported in a recent paper which discusses the rationale and evidence for the use of metformin in obese children and young people at high risk of T2DM [17]. The available evidence indicates that, in the short term, administration of metformin in addition to lifestyle modification is relatively effective in reducing BMI and hyperinsulinemia among obese adolescents, without related morbidity, and displays an acceptable safety pattern. Nevertheless, its long-term impact is unknown [18, 19]. In fact, metformin appears to be moderately efficacious in reducing BMI and IR in hyperinsulinemic obese children and adolescents in the short term. Larger, long-term studies in different populations are needed to establish its role in the treatment of overweight children [20]. Some authors concluded that a limited period of such a treatment may help weight control and might be used to encourage those children who have been refractory to weight loss for continuing the non-pharmacological programs. These results should be confirmed in studies with a longer follow-up period [21, 22]. The combination of diet and exercise followed by metformin in the early phase of “IR” may reduce or delay both atherosclerosis and its complications associated with diabetes. Prevention therapy must begin much earlier than clinical diagnosis of diabetes, aiming to initially lower blood insulin levels or IR [23]. A recent metanalysis shows that metformin provides a statistically significant, but modest reduction in BMI when combined with lifestyle interventions over the short term in obese children. In the context of other options for treating childhood obesity, metformin has not been shown to be clinically superior [24]. Metformin is a drug effective in reducing weight in a naturalistic outpatient setting in insulin-sensitive and insulin-resistant overweight and obese patients [25]. The results of an old study demonstrated that metformin contributes to a reduction in body weight, body fat mass and waist circumference, improves insulin sensitivity and decreases basal, total and stimulated insulin secretion in obese subjects. Thus, metformin appears to be an effective and well-tolerated drug in the treatment of obesity in subjects with normal glucose tolerance, it improves not only the BMI, but also the body composition reducing fat mass [26]. A significant decrease in body weight, BMI, percentage body fat, the sum of saturated fatty acids in serum phospholipids and increase in insulin sensitivity index were observed following a 20-week treatment. These changes did not differ significantly between the groups. The results of a recent study suggest that treating obese adolescents with IR using metformin for 3 months is an option for patients without response to traditional lifestyle change because metformin decreases inflammatory activity, which is an etiological factor in cardiovascular disease development [27]. Metformin has no effect on blood pressure and blood glucose levels, but it does reduce total cholesterol, abdominal obesity and C-reactive protein levels in obese hypertensive patients without diabetes. [28]. Metformin trials are heterogeneous, but one large, good-quality trial showed a weight loss of 2.3 kg more in the intervention group. Weight loss treatments did not improve health outcomes, but they were not homogenous in treatment methodology and most trials were not powered for outcomes such as death and cardiovascular events. Weight loss treatment resulted in a reduction in diabetes incidence in two large, good-quality behavioral-based trials of diabetes prevention. Behavioral-based treatment showed small positive effects on blood pressure [29]. Moreover, metformin may improve sense of satiety and decrease anxiety about food in some individuals with Prader–Willi syndrome and early morbid obesity. Positive response to metformin may depend on the degree of hyperinsulinism and glucose intolerance. Anyway, the results of this pilot study require further investigation [30]. Energy restriction rather than metformin treatment appears to be responsible for the observed changes. The associations previously found in diabetics between insulin sensitivity and phospholipid fatty acids may not be mediated by metformin [31]. The benefic effect of alpha-lipoic acid in reducing IR in obese patients is controversial: there are positive results in animals and in vitro [32], but these results were not confirmed in humans [33].

### Polycystic ovary syndrome (PCOS)

Polycystic ovary syndrome (PCOS) is the most common endocrinopathy among women in reproductive age, with a prevalence of 5–15 % of the general population [34, 35]. PCOS is characterized by menstrual disturbances, anovulatory infertility and high levels of male hormones which appear in the early reproductive years; obesity is often associated with these signs and symptoms [36]. The pathogenesis of PCOS is not completely understood. It can be defined as a multifactorial endocrine disorder in which multiple genes are involved. Based on the clustering of cases in families, PCOS is considered a heritable disorder [37]. Genetic influences are suggested by a high prevalence of PCOS or its features among first-degree relatives [38]. In addition, in monozygotic twins, there is a greater concordance rather than in dizygotic twins [39]. However, the mode of inheritance is not clear. It is also now accepted that some environmental factors may influence the clinical expression of the disorder: the presence of obesity, for example, often contributes to accelerate the onset of the disease in women predisposed to develop it or aggravates its clinical presentation in women who have a mild form of the syndrome. Anyway, several studies have clearly shown that IR plays a key role in the pathogenesis of PCOS and of its metabolic and cardiovascular abnormalities [40, 41]. Burghen found higher insulin concentrations, at basal state and under glucose stimulation, in women with PCOS rather in women without PCOS of the same age and weight [42]. Other data suggest that affected women have a higher IR [43], independently by obesity degree and by presence of impaired glucose tolerance [44]. In PCOS, IR seems to be related to an excessive constitutive phosphorylation of serine β-subunit of the insulin receptor, by a serine–threonine kinase. The defect is selective, involving the metabolic effects of insulin, but not mitogenic one [43]. Approximately 50 % of PCOS patients, both obese and slim, display IR associated with hyperinsulinemia [45]. These women have a significantly higher prevalence of biochemical and hirsutism than women with non-insulin-resistent PCOS [41]. Furthermore, they develop more often menstrual disturbances, have a lower ovulation rate and are more often resistant to clomiphene treatment [46]. Anyway, PCOS is not diagnosed in all obese women with IR [40]. Probably in same women, IR should be considered an important factor in pathogenesis of PCOS, but not the primary defect causing this disease. Considering the key role of IR in pathogenesis of PCOS and the coexistence of reproductive and cardiometabolic abnormalities in the context of the same disease, therapeutic management of affected women is changed in the last years. Insulin-sensitizing drugs (metformin, pioglitazone, d-chiro-inositol) have been introduced as a therapeutic option in PCOS, targeting reproductive and cardiometabolic abnormalities on the basis of its action on the reduction of glucose levels and the attenuation of IR [13]. Metformin exerts its principal metabolic action and especially its gluco-regulatory action upon the liver; anyway, it has pleiotropic actions on several other tissues affected by IR such as the skeletal muscles, the adipose tissue, the endothelium and the ovary. The increase in insulin sensitivity occurs in women with PCOS without diabetes [47]. Metformin has a direct effect on steroidogenesis in ovarian cells and an indirect effect due to the alleviation of insulin excess acting upon the ovary. Studies on cultures of ovarian theca cells show that metformin reduces CYP 17 activity directly and through reduction of insulin levels [48]. Hence, the primary trend of theca cells to increase synthesis and secretion of androgens is reduced using metformin in PCOS disease: it promotes the suppression of Δ-4A production through reduction of CYP 17 lyase activity rather than CYP 17 hydroxylase activity [47, 49]. Moreover, a decrease of insulin levels promotes the reduction of activity of other steroidogenetic enzymes in these cells, such as 3b-HSD and P450scc. Metformin has shown to reduce basal and FSH-stimulated progesterone and estradiol production both in rat than in cultured human granulosa cells from women with or without PCOS [50] and metformin treatment was associated to a decrease of steroidogenesis and aromatase protein expression and to a inhibition of granulosa cell proliferation [51]. In PCOS patients, long-term metformin treatment, may increase ovulation, improve menstrual cyclicity [52, 53], and reduce serum androgen levels [54]. A recent review [55] summarizes the effects of long-term therapy with metformin in women with PCOS. Metformin improves both ovulation and pregnancy rates but it does not improve live birth rates and miscarriage. The effect of metformin treatment on ovulation and pregnancy rate is higher in non-obese PCOS patients. The improvement of pregnancy rate is linked to an improvement in menstrual pattern. Metformin has a significant effect in reducing fasting insulin levels in the non-obese women and it reduces serum testosterone concentrations. Metformin has no effect on fasting glucose levels, serum lipid profiles and on anthropometric parameters as BMI and waist circumference (WC); reduction in blood systolic pressure has been observed. Metformin can delay the progression of glucose intolerance in women with PCOS [56]. A recent consensus anyway does not recommend its routine use in the treatment of women with anovulatory PCOS. The lifestyle modification should be the first-line management approach and form an integral part of managing obese PCOS women, with metformin being less effective in these patients [57]. Some authors [58] suggest that clomiphene remains the first-line ovulation induction agent and metformin may be added as an alternative ovulation induction agent for clomiphene-resistant obese women with PCOS. Furthermore, metformin may be added in patients with glucose intolerance or T2DM who do not respond adequately to calorie restriction and lifestyle changes. Even though it has been shown that metformin treatment reduces testosterone levels, the effects on hirsutism and acne described in clinical trials are modest [54]. Hence, metformin is unlikely to completely replace combined oral contraceptives as a first-line therapy for hirsutism.

In respect to the use of other insulin-sensitizing drugs, few trials are available. Use of pioglitazone in women with PCOS is associated with an improvement of ovulation rate and menstrual pattern, but not of endocrine parameters such as testosterone, insulin, lipid profile and glucose levels. Anyway, recent consensus recommended to avoid use of thiazolidinediones in management of PCOS women [58].

Inositol is described as a second messenger system that may exert an insulin-like effect on metabolic enzymes [59]: for this reason, it has been used as insulin-sensitizing agent in women affected by PCOS. Some studies reported an improvement in insulin sensitivity and ovulatory function in the women treated, both in the isoform d-chiro-inositol [60], and in the isoform myo-inositol [61]. Anyway a meta-analysis [55] of some studies shows any improvement in ovulation rate, any effects on BMI and WC and any effects on testosterone, fasting glucose and insulin levels and lipids profile. So, its use is not recommended in PCOS women.

Since IR is also present in lean women with PCOS, several studies have suggested that an increased oxidative stress could concur to the presence of IR in these patients [62]. The use of biological antioxidants has been therefore proposed in the treatment of PCOS women. Alpha-lipoic acid (ALA), which is synthesized by the liver, is a biological antioxidant and natural cofactor of mitochondrial dehydrogenase complexes. In vitro studies have provided evidence that ALA can improve insulin sensitivity, activating two important molecules of the insulin signaling pathway-insulin receptor substrate-protein and phosphatidylinositol 3-kinase [63]. Some authors observed an improvement in insulin sensitivity with ALA therapy in these women despite the absence of severe IR [64]. Probably ALA has also been involved in AMPK activation such as metformin and thiazolidinediones [65]. Further studies are necessary to understand the molecular pathways of ALA and its possible use in the management of lean insulin-resistant women with PCOS.

### NAFLD

Non-alcoholic fatty liver disease (NAFLD) is the most common liver disorder worldwide. NAFLD is a general diagnosis which encompasses a spectrum of pathological processes ranging from non-alcoholic fatty liver (NAFL or simple steatosis) to non-alcoholic steatohepatitis (NASH), fibrosis, cirrhosis, and hepatocellular carcinoma [66]. The pathophysiology is thought to be a two-step process characterized by the liver accumulation of triglycerides and free fatty acids induced by IR and the subsequent hepatocytes’ injury due to the oxidative stress and to the release of proinflammatory cytokines [67]. IR represents the main pathogenic factor underlying these metabolic disorders. Although the molecular mechanism is still unclear, free fatty acids and triglycerides metabolites have been suggested to act directly or via toll-like receptors 2 and 4, inducing endoplasmic reticulum stress, mitochondrial dysfunction, ROS production, impairment of liver protein metabolism, inhibition of insulin signaling and activation of several inflammatory pathways [68]. Moreover, steatosis determined the release of factors called hepatokines which play the role of key mediators in the pathogenesis of local and systemic inflammation and in the further impairment of peripheral insulin sensitivity [69]. The subsequent chronic inflammatory condition induces the progression from steatosis to more advance stages of liver damage [70]. Epidemiological studies reported a significant association between NAFLD and conditions associated with IR, such as obesity, T2DM, hyperlipidemia, hypertension, and PCOS [71]. Due to the increased risk of cirrhosis and hepatocellular carcinoma, NAFLD is expected to become the leading indication for liver transplant by 2020 [72]. Recent evidences suggest that NAFLD may represent an independent cardiovascular risk factor related to a significant increased mortality compared to the general population [70]. Considering the key role of IR in pathogenesis of NAFLD, insulin-sensitizing drugs (such as metformin, thiazolidinediones) have been investigated as a therapeutic option.

Beneficial effects of metformin on NAFLD may be related to the adenosine monophosphate-activated protein kinase (AMPK) pathway. Upon activation, AMPK stimulates activation of catabolic and inhibition of ATP-dependent anabolic processes. Furthermore in the liver, AMPK reduces gluconeogenesis by the phosphorylation of CREB-binding protein (CBP) and the dissociation of the gluconeogenic CREB–CBP–TORC2 transcriptional complex [67]. Moreover, metformin decreases cholesterol and fatty acid synthesis and promotes malonyl-CoA carboxylase activity through the inhibition of acetyl-CoA carboxylase (ACC) and 3-hydroxy-3-methylglutaryl (HMG)-CoA reductase [73]. More recently, data in vivo and in vitro suggest that metformin prevents hepatic steatosis by regulating the expression of cellular mediators of lipid storage [74]. However, data about improvement in liver enzymes and hepatic histology during treatment with metformin in patients with NAFLD/NASH are controversial. Several open-label clinical studies [75–84] supported the beneficial effects on serum aminotransferases levels [75], and on IR markers [75, 76, 80–84] of NAFLD/NASH patients treated for at least 6 months with metformin (dose ranging 1.4–2.0 g/day) alone or in association with vitamin E [94] or lifestyle intervention [75, 78, 84]. Only three open-label studies have found no benefits of metformin treatment on aminotransferase levels and IR markers [85–87]. However, only few studies shown an improvement in liver histology [77, 78, 81, 83]. These results have not been confirmed by the larger randomized clinical trials [76, 82, 85, 88] comparing metformin and lifestyle intervention with lifestyle changes alone. Results in pediatric population were similar to those of adults, thus supporting positive effects on biochemistry liver profile and metabolic parameters, but not on liver histology. The randomized multicenter and placebo-controlled TONIC trial (treatment of nonalcoholic liver disease in children) failed to demonstrate in a large sample of children the superiority of metformin to placebo in attaining a sustained reduction of amino transferase levels and significant improvement in histological features [89].

A recent systematic review and meta-analysis concluded that treatment with metformin for 6–12 months together with lifestyle intervention did not show any significant improvement in aminotransferases circulating levels or liver histology [90]. Based on these results, the current practice guidelines by the American Association for the Study of Liver Diseases, American College of Gastroenterology, and the American Gastroenterological Association do not recommend metformin as a treatment for NAFLD [91].

Thiazolidinediones (TZD) which are selective ligands of the nuclear transcription factor peroxisome proliferator-Υ (PPAR-Υ) have been largely investigated in patients affected by NAFLD/NASH. Following the activation of PPAR-ϒ, these drugs can up-regulate fatty acid disposal and ameliorate tissue insulin sensitivity. In addition, TZD promote fatty acid uptake and storage in adipose tissue, sparing other insulin-sensitive tissues, such as skeletal muscle and the liver, thus reversing the down-regulation of IRS-1 which leads to IR [92]. Results coming from uncontrolled open-label trials [82, 93, 94] were confirmed by larger controlled trials. A double-blind, placebo-controlled study performed on impaired glucose-tolerant (IGT)/T2DM patients, has shown that pioglitazone treatment (30–45 mg/day) for 6 months significantly ameliorates aminotransferase levels (by 50 %), steatosis (by 54 %), IR markers (by 48 %), liver inflammation and necrosis, but not fibrosis [95]. A similar trial conducted in non-diabetic individuals affected by NASH revealed a significant improvement in liver fibrosis together with liver enzymes and histological necro-inflammatory markers reduction after 12 months [96]. The PIVENS study, a multicenter, placebo-controlled trial analyzed the efficacy of pioglitazone (30 mg/day) versus placebo and vitamin E in 247 non-diabetic patients with biopsy-proven NASH. The primary endpoint defined as an improvement in histological findings accordingly with the NAFLD Activity Score [97] was achieved by 34 % of pioglitazone-treated patients and 43 % of vitamin E-treated individuals in comparison to 19 % of control subjects. Moreover, resolution of NASH, a secondary endpoint has been detected in a significantly higher number of patients receiving pioglitazone versus placebo, despite a significant body weight increase [98]. A recent meta-analysis including 4 randomized clinical trials demonstrated that pioglitazone significantly improves steatosis, hepatic flogosis, necrosis and liver fibrosis [92].

Based on these results, the current practice guidelines by the American Association for the Study of Liver Diseases, American College of Gastroenterology, and the American Gastroenterological Association suggest to evaluate the use of pioglitazone to treat steatohepatitis only in patients with biopsy-proven NASH, noting that long-term safety and efficacy of pioglitazone in non-diabetic patients is not completely established [91].

## Conclusions

Certainly many studies suggested the benefit of the treatment of insulin resistance in many clinical conditions besides type 2 diabetes and it is desirable to have in the future more indications overall for metformin. In particular, a new formulation of metformin extended-release (ER) is now available with different formulation in the base on the different countries. This was reached using a Gelshield diffusion system that gives metformin a slower absorption than IR with a maximum plasma concentration of 7 versus 3 h. Metformin ER releases the active drug through hydrated polymers which expand safe uptake of fluid, prolonging gastric transit and slowering drug absorption in the upper gastrointestinal tract [99, 100]. In addition, Metformin ER causes a small, but statistically significant decrease in BMI when added to a lifestyle intervention program in obese adolescents [101].
